# Early Outcomes of Minimally Invasive Anterior Longitudinal Ligament Release for Correction of Sagittal Imbalance in Patients with Adult Spinal Deformity

**DOI:** 10.1100/2012/789698

**Published:** 2012-12-10

**Authors:** Armen R. Deukmedjian, Elias Dakwar, Amir Ahmadian, Donald A. Smith, Juan S. Uribe

**Affiliations:** Department of Neurosurgery and Brain Repair, University of South Florida, 2 Tampa General Circle, 7th Floor, Tampa, FL 33606, USA

## Abstract

The object of this study was to evaluate a novel surgical technique in the treatment of adult degenerative scoliosis and present our early experience with the minimally invasive lateral approach for anterior longitudinal ligament release to provide lumbar lordosis and examine its impact on sagittal balance. *Methods*. All patients with adult spinal deformity (ASD) treated with the minimally invasive lateral retroperitoneal transpsoas interbody fusion (MIS LIF) for release of the anterior longitudinal ligament were examined. Patient demographics, clinical data, spinopelvic parameters, and outcome measures were recorded. *Results*. Seven patients underwent release of the anterior longitudinal ligament (ALR) to improve sagittal imbalance. All cases were split into anterior and posterior stages, with mean estimated blood loss of 125 cc and 530 cc, respectively. Average hospital stay was 8.3 days, and mean follow-up time was 9.1 months. Comparing pre- and postoperative 36′′ standing X-rays, the authors discovered a mean increase in global lumbar lordosis of 24 degrees, increase in segmental lumbar lordosis of 17 degrees per level of ALL released, decrease in pelvic tilt of 7 degrees, and decrease in sagittal vertical axis of 4.9 cm. At the last followup, there was a mean improvement in VAS and ODI scores of 26.2% and 18.3%. *Conclusions*. In the authors' early experience, release of the anterior longitudinal ligament using the minimally invasive lateral retroperitoneal transpsoas approach may be a feasible alternative in correcting sagittal deformity.

## 1. Introduction

 Many factors are involved in the surgical management of adult spinal deformity, including maintenance of coronal and sagittal balance, as well as spinopelvic harmony [1–5]. Adult spinal deformity (ASD) is believed to develop because of asymmetrical degeneration of discs, osteoporosis, and vertebral body compression fractures [6]. Presenting symptoms of this condition primarily includes radiculopathy, chronic low back pain and neurogenic claudication caused by concurrent spinal stenosis [7, 8].

 Studies by Schwab et al. [9] and Glassman et al. [10] have demonstrated that in the treatment of congenital and acquired deformity, correction of sagittal alignment to an SVA <5 cm leads to improved clinical outcomes. One of the multiple limitations of MIS techniques is that up till now they have been unable to improve sagittal balance significantly [11]. Sagittal imbalance is traditionally managed with posterior shortening osteotomies, anterior lengthening maneuvers, or both. Classically, closing wedge osteotomies include Smith-Peterson osteotomy (SPO), pedicle subtraction osteotomy (PSO), and vertebral column resection (VCR), which have been reported to have a 41% complication rate in ASD [12, 13]. Major complications in revision adult deformity surgery were reported by Cho et al. to be 34% in a retrospective review of 141 patients [14]. Sectioning the anterior longitudinal ligament (ALL) via the minimally invasive (MIS) lateral transpsoas approach with placement of a hyperlordotic cage has been proposed as an alternative to open traditional osteotomy for correction of sagittal plane deformity [15, 16]. Up to this point, literature on this subject has been scarce, and to the authors' knowledge, the safety and early outcomes of this procedure have not yet been examined. In this paper, we describe our early clinical experience with ALL release (ALR) for the purpose of increasing lumbar lordosis and improving sagittal misalignment.

## 2. Materials and Methods

 We retrospectively reviewed a prospectively acquired database of all patients with adult thoracolumbar degenerative deformity treated with an ALR via the minimally invasive, lateral retroperitoneal transpsoas interbody fusion (MIS LIF) at our institution. Parameters reviewed include patient demographics, preoperative/postoperative evaluations, spinopelvic parameters, sagittal vertical axis (SVA), procedure performed, operative time, blood loss (EBL), length of hospital stay, and complications. In order to quantitate early outcome measures, we compared preoperative and postoperative scores on the visual analogue scale (VAS) and the Oswestry Disability Index (ODI). These linear scales provide a percentage from 0 to 100%, with 0 representing no pain or disability and 100 representing complete disability and pain. Postoperative followup took place at weeks 2, 6, 12, 24, and 52 when available. If patients did not show up for a scheduled follow-up appointment, VAS and ODI scores were obtained over the telephone.

All patients in the cohort presented with mechanical back pain with or without radicular pain refractory to at least 12 months of nonoperative management and were in global sagittal malalignment. According to the Lenke-Silva classification for ASD, patients fell into levels 2, 3, 4, or 5 and were managed with a MIS version of their operative schema [17]. All patients with sagittally maligned ASD were evaluated for ALR although not all were candidates for this procedure. Patients with adolescent idiopathic scoliosis (AIS) or scoliosis secondary to neuromuscular conditions were excluded from the study.

## 3. Operative Technique

**Figure 1:**
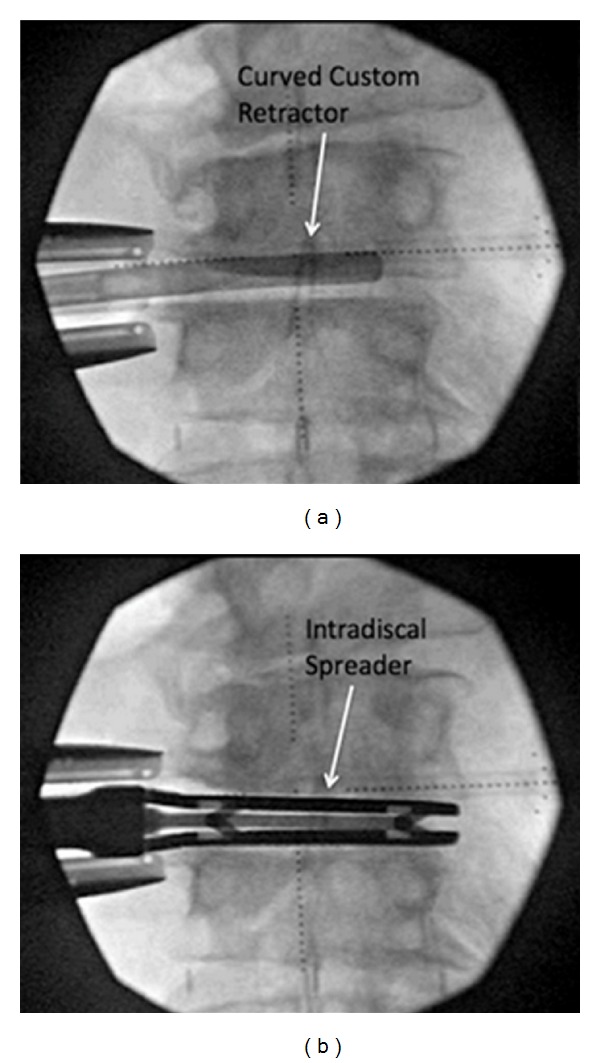
Intraoperative anterior-posterior radiographs demonstrating the curved retractor anterior to the disc space during dissection of the sympathetic plexus off the great vessels (a), and the intradiscal spreader in the disc space used to break the contralateral ALL remnant rather than blindly incising (b).

**Figure 2:**
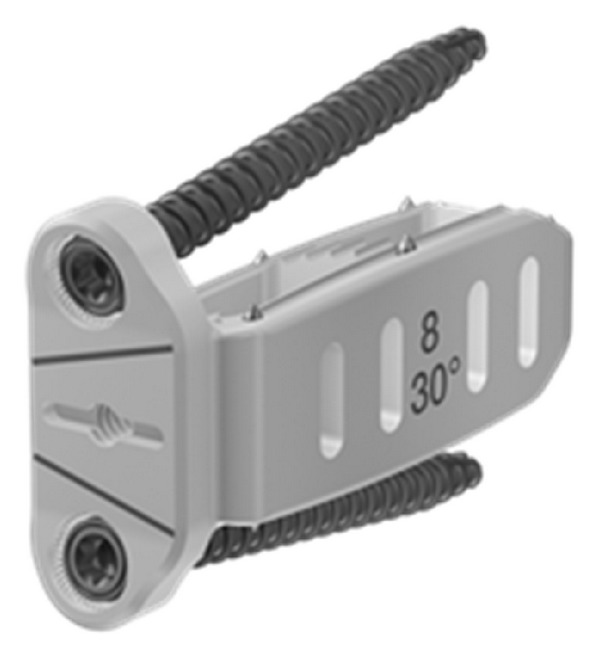
Photograph demonstrating the 30-degree hyperlordotic cage with attached screws to prevent ventral migration into the peritoneum.

Prior to attempting this procedure in vivo, in order to minimize complications, we strongly encourage cadaveric dissection and a review of the literature focusing on the safe zones of the lateral approach [18–21]. In addition to the anatomical nuances regarding the lateral approach previously described, the anatomy of the ALL from the perspective of the lateral transpsoas approach is described by Deukmedjian et al. in a recent study [22]. The surgical procedure consisted of a variation of the previously described technique for the lateral retroperitoneal transpsoas approach to the lumbar spine [23]. After performing the discectomy with careful attention to endplate preparation, a slight curved custom retractor/dissector was gently passed along the anterior edge of the ALL and positioned between the large vessels/sympathetic plexus and the ventral aspect of the disc (Figure 1). Dissecting dorsal to the great vessels is the key step in this procedure, and although we have had no catastrophic complications, it would be possible at this step. Although there is a plane ventral to the ALL, a common pitfall we found during cadaveric dissection was to mistake the sympathetic plexus for the lateral edge of the ALL, which would lead to sectioning of the plexus. We are currently evaluating our patients for clinical ramifications of this for a future study. At this point, using a custom ligament blade and intradiscal distractor, the ALL was sectioned in a sequential fashion, easing the curved retractor across to the contralateral side of the disc space. With complete ALL sectioning, there was immediate mobilization and “fish-mouthing” of the adjacent vertebral body endplates. An appropriate sized hyperlordotic poly-ether-ether-ketone (PEEK) cage was selected at this point (CoRoent XL-Hyperlordotic, NuVasive, Inc., San Diego, CA, USA). These cages were packed with allograft (Osteocell, NuVasive, Inc., San Diego, CA, USA) and anchored to the adjacent VB with one or two screws to prevent ventral migration into the peritoneal cavity and loss of indirect decompression (Figure 2). In each case, pedicle screws were placed posteriorly to stabilize the construct, most commonly using a percutaneous technique.

## 4. Results

**Figure 3:**
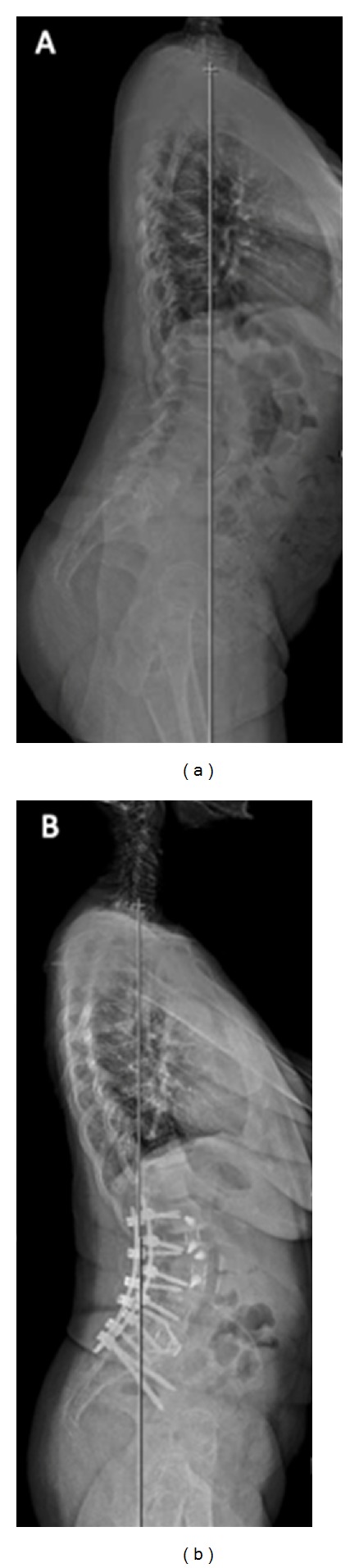
(a) Pre- and (b) postoperative standing 36 inch lateral radiographs demonstrating improvement in sagittal vertical axis after two-level ALR plus multilevel MIS LIF with open posterior instrumentation.

From 2010 to 2012, 7 patients (4 women, 3 men) underwent a MIS lateral retroperitoneal transpsoas approach to release the ALL in the treatment of sagittal imbalance in patients with ASD (Table 1). The mean patient age was 64.7 years (range 58–71). All patients successfully had 30-degree hyperlordotic cages placed in conjunction with standard cages at the other lumbar levels for anterior column support and interbody fusion (Figure 3). ALR was performed at 11 levels in the 7 patients (average 1.6 per patient), while a total number of 28 interbody fusions (average 4 per patient) were performed. 51 levels were fixated during a second stage with pedicle screws (average 7.3 per patient), all of which were done using the percutaneous technique except for one patient who had a revision surgery. 

Average EBL was 125 cc for stage I and 530 cc for stage II, and the average length of hospital stay was 8.3 days (there was a minimum of 5 days between stage I and II). No patients required a blood transfusion and there were no catastrophic complications to report in our cohort. There were no durotomies, blood vessel or bowel injuries, and no patient had lasting postoperative weakness. One patient, however, did have a superficial wound infection in the lateral incision that was treated successfully with a wound washout and a short course of intravenous antibiotics.

**Figure 4:**
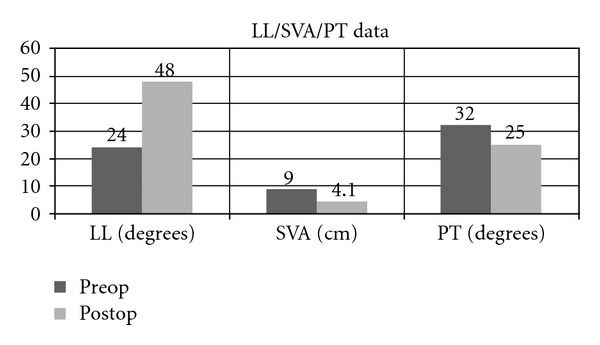
Graph demonstrating pre- and postoperative values for lumbar lordosis (LL), sagittal vertical axis (SVA), and pelvic tilt (PT) in our cohort.

During postoperative radiographic assessment, it was noted that there was an average increase in global and segmental lumbar lordosis of 24 and 17 degrees, respectively (Table 2). Overall sagittal balance improved by 4.9 cm, as measured on the SVA, going from 9 cm to 4.1 cm. Pelvic tilt (PT) decreased by an average of 7 degrees, from 32 to 25 degrees (Figure 4). 

**Figure 5:**
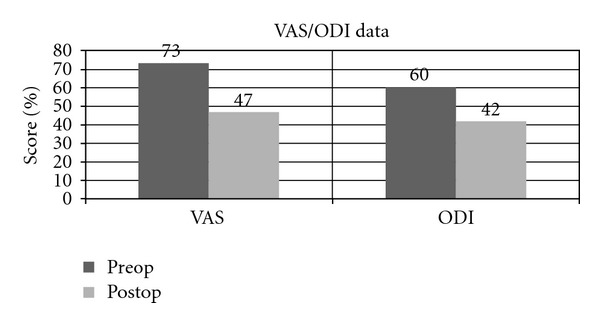
Graph demonstrating pre- and postoperative values for visual analogue scale (VAS) and Oswestry Disability Index (ODI).

VAS and ODI scores were used as outcome measures, and average time from surgery to filling out the latest questionnaire was 9 months. VAS and ODI scores improved an average of 26.2% and 18.3%, respectively. VAS scores went from an average of 73% to 46.8% after surgery, while the ODI scores improved from an average of 60% to 41.7% after surgery (Figure 5). 

## 5. Discussion

With a growing elderly population demanding a longer active lifestyle, the impetus has been placed on spine surgeons to use innovations in technology to provide less invasive solutions to increasingly complex spinal deformities. Asymmetric degeneration of disc spaces in the thoracolumbar/lumbar spine is believed to be one of the causes that result in adult degenerative scoliosis/deformity. Symptoms of this class of spinal deformity may range from relatively asymptomatic to axial or radicular pain in 90% of patients [7, 8]. In many cases, patients with ASD are opting for surgical intervention when conservative measures fail. Traditional goals of adult deformity surgery are correction of coronal and sagittal balance and obtaining a solid fusion. However, treatment of adult spinal deformity is constantly evolving, and radiographic goals such as pelvic tilt <25 degrees and LL = PI ± 9 degrees have been established [4, 9, 24]. For the purposes of this study, we focus here on improving sagittal balance to an SVA <5 cm.

Although the importance of sagittal plane deformity has been well studied, especially in the context of flat back syndrome, we now have a guideline to keep sagittal balance, or SVA less than 5 cm to optimize clinical outcomes [25–28]. Sagittal plane correction is traditionally accomplished through posterior shortening techniques, such as a Smith-Peterson (SPO) or pedicle subtraction osteotomy (PSO), which, although effective, may be associated with significant morbidity [29–31]. Another option is the release of the anterior longitudinal ligament. Although not a new concept, it is relatively infrequently practiced because of significant approach-related morbidity [32–36]. Recently, however, the lateral retroperitoneal/retropleural approach to the thoracic and lumbar spine has provided spine surgeons with another, less invasive option in scoliosis surgery [37]. It has been shown previously that minimally invasive spine surgery results in less blood loss, reduced muscle dissection/trauma, shorter hospital stays, and faster mobilization and recovery after surgery [38]. 

### 5.1. Technical Aspects of MIS ALL Release

In this study we describe our experience with MIS ALR, and through our results show that it is not only a safe option but also one that provides significant improvements in sagittal balance with low morbidity. As with all new MIS techniques, there is a steep learning curve, and the most important factor is understanding the procedure and the surrounding anatomy. In addition to the usual risks associated with the lateral approach, unique perils associated with ALR include great vessel injury and damage to the sympathetic plexus [22]. However, the anatomical dissection plane is ventral to the ALL and dorsal to the sympathetic plexus and great vessels, making injury less likely. In addition, we avoid electrocautery and use a modified 15 blade to cut the ALL to minimize damaging surrounding tissues. Placing the patient in the left lateral decubitus position also allows better control of the inferior vena cava. In order to perform the final release of the ALL, use of the intradiscal distractor may avoid blind sectioning of the ligament on the contralateral side. After completion of the ALR, placing the hyperlordotic cage in the middle of the disc space may increase the disc height and provide indirect foraminal decompression. The cage is then secured into place with a screw to avoid anterior migration into the peritoneum.

### 5.2. Clinical Implications on MIS ALL Release

Although the average follow-up time in our series is only 9 months, we believe that this is adequate for the purposes of identifying the safety and efficacy of this procedure since the main results and complications occur during the immediate postoperative period. Ongoing analysis of these patients is being performed with the purpose of a future study to evaluate if the sagittal balance is maintained.

Short-term complications include vascular and neural injuries, while long-term complications include subsidence, pseudarthrosis, and adjacent segment failure, which are not unique to this surgery.

Length of hospital stay in our cohort was 8.3 days, similar to the 7.9 days reported by Schwab et al. in the group of patients without complications [39]. In our practice, surgery for adult deformity is generally done in two stages. The lateral approach for placement of interbody cages as well as sectioning the ALL if necessary is done during the first stage, and posterior fixation with either percutaneous or open pedicle screw placement is performed in the second stage. We recommend breaking large deformity cases into two stages for various reasons, including ease on patient in terms of operating time and related complications and ease on surgeon preventing long and difficult procedures. Another important factor is the ability to reassess the patients' spinopelvic parameters between stages to customize planning for the posterior procedure and assess need for hybrid constructs including an MIS SPO or open laminectomy/instrumentation versus percutaneous screw fixation.

Mean blood loss in our cohort during stage 1 was 125 cc, and during stage 2 was 530 cc, with a total of 655 cc. The International Spine Study Group recently published a study demonstrating that greater intraoperative blood loss (>2.4 L) is a major risk factor for perioperative complications [39]. This is another benefit of correcting sagittal balance through a minimally invasive approach. The global lumbar lordosis in our group improved by 24 degrees, from 24 to 48 degrees, while segmental lordosis at the levels where an ALR was performed improved an average of 17 degrees. Given that the SVA improved by 4.9 cm, from 9 to 4.1, and within the range established by Lafage et al., we expect postoperative outcome scores to be improved [40, 41]. This is in fact the case, as VAS and ODI scores improved by 26 and 18%, respectively. Another likely contribution to the overall patient improvement is that the pelvic tilt improved on average by 7 degrees, from 32 to 25 degrees, also within the recommended range of less than 25 degrees.

### 5.3. Complications

All techniques for restoration of sagittal balance have significant risk of complications and are technically challenging [14, 39]. We attribute our low rate of complication to multiple factors, the most important of which is understanding the regional anatomy. Before attempting an ALR which has a steep learning curve, we spent time in cadaveric dissection in the lab, isolating the sympathetic plexus as it runs along the anterolateral border of the lumbar vertebral bodies in order to determine if there was a plane between the ALL and the sympathetic plexus/great vessels. Only when it was determined to be feasible in cadaveric specimens were we willing to try it in our patients [15, 22]. After performing MIS LIF for five years on over a thousand levels and multiple dissections and publications, we felt confident in our ability to perform an ALR. As of yet there are no cases of subsidence, which is a potential complication with hyperlordotic cage placement.

In our cohort of 7 patients, we had no complications except for a superficial wound infection treated successfully with a wound washout and short course of intravenous antibiotics.

### 5.4. Limitations

During the dissection there is no adequate proximal and distal control of the great vessels. In case of injury to the aorta or inferior vena cava, the surgeon would likely need to extend the skin incision anteriorly and perform direct compression of the vascular structures followed by proximal and distal control, followed by direct repair of the defect. 

The low number of patients (*n* = 7) in our cohort was a potential limitation in this study; however, we believe that it was adequate to demonstrate our results as well as the feasibility of this technique. At this point we must again stress the importance of understanding the surrounding anatomy if attempting an ALR, as its true safety lies in the hands of the surgeon performing the procedure. The mean follow-up time of 9 months is also slightly below the 2-year standard, but in the context of a new surgical technique is sufficient for our goals. We believe that this technique requires further study to be able to draw general conclusions, and we will continue to follow these patients to assess long-term outcomes.

## 6. Conclusions

Sagittal imbalance is a causative factor of clinical impairment and is of great concern to spine surgeons. It can be managed through anterior lengthening procedures and posterior shortening techniques. Both are historically associated with significant morbidity. Our early experience using the MIS lateral retroperitoneal transpsoas approach to release the ALL and place a hyperlordotic cage shows that this approach may give up to 17 degrees of segmental lordosis and may be a feasible alternative to more traditional approaches such as posterior osteotomies.

## Figures and Tables

**Table 1:** Demographic and surgical data of cohort (*n* = 7).

Case	Age/sex	DOS	Level(s) of ALR	Interbody levels	Fixation levels	Blood loss (1st stage/2nd stage)	Complications
1	67 F	8/13/10	L2/3	L1-S1	T10-iliac	100/300	—
2	67 M	2/8/12	T12/L1 L2/3 L3/4	T12-S1	T10-S1	20/1100	—
3	71 F	10/26/10	L1/2	L1-5	T10-L5	100/500	—
4	69 F	3/16/11	L2/3	L2-5	L2-5	10/100	—
5	62 M	5/18/11	L2/3 L3/4	L1-5	T12-L5	100/300	—
6	58 F	2/16/11	L2/3 L3/4	L2-S1	L2-iliac	500/1100	Superficial infection
7	59 M	11/16/11	L3/4	L1-S1	T11-S1	50/300	—

**Table 2:** Pre- and postoperative spinopelvic parameters and outcome measures of the cohort.

Case	Preop LL	Postop LL	Preop SVA	Postop SVA	Preop PT	Postop PT	Preop VAS	Postop VAS	Preop ODI	Postop ODI
1	10	37	18	15	38	35	75	50	56	42
2	28	61	10	1.5	48	25	55	28	54	34
3	22	46	7.5	5.5	43	34	90	63	92	66
4	19	39	3.2	2.8	31	26	68	65	40	34
5	33	51	10.5	0	35	28	43	18	22	18
6	37	61	7	0.5	18	18	100	83	86	42
7	20	40	6.5	4	10	7	80	20	70	56

Mean	24	48	9	4.1	32	25	73	47	60	42
